# Daily adaptive radiotherapy in head and neck squamous cell carcinoma can negatively impact organ at risk dosimetry

**DOI:** 10.1016/j.ctro.2026.101205

**Published:** 2026-05-30

**Authors:** Hedda Enocson, André Haraldsson, Per Engström, Sofie Ceberg, Maria Gebre-Medhin, Gabriel Adrian, Per Munck af Rosenschöld

**Affiliations:** a Medical Radiation Physics, Department of Clinical Sciences Lund, Lund University, Lund, Sweden; b Radiation Physics, Department of Hematology, Oncology, and Radiation Physics, Skåne University Hospital, Lund, Sweden; c Oncology, Department of Clinical Sciences Lund, Lund University, Lund, Sweden; d Department of Hematology, Oncology, and Radiation Physics, Skåne University Hospital, Lund, Sweden

**Keywords:** Adaptive radiotherapy, Head and neck cancer, Protocol-driven adaptation, Dose tracking

## Abstract

**Purpose:**

Adaptive radiotherapy (ART) accounts for anatomical changes during treatment and may improve target coverage and spare organs-at-risk (OARs) in head and neck squamous cell carcinoma (HNSCC). This study aimed to develop a protocol defining dose evaluation criteria for target volumes and OARs to assess the need for ART. The protocol was applied to compare compliance between daily-ART and non-ART plans with varying CTV-PTV-margins.

**Materials and methods:**

A protocol defining dose criteria for target and OAR volumes was developed and used to evaluate ART in a retrospective cohort of 80 patients (2,170 fractions) treated with radiotherapy to 60.0–68.0 Gy (2 Gy/fraction). Synthetic CTs were generated from daily kVCTs and contours propagated using deformable image registration. Delivered doses (no-ART) and daily-ART treatments using 5 mm and 2 mm PTV plans were simulated. All daily distributions were evaluated using the protocol.

**Results:**

With 2 mm margins, daily-ART significantly reduced protocol violations compared to no-ART(7% vs 20.3%, p < 0.001), while no significant difference was observed with 5 mm margins(15.1% vs 17.3%, p > 0.8). For 2 mm margins, daily-ART resolved 610 violations but introduced 152 new violations. With 5 mm margins, 391 violations were resolved, while 472 violations were introduced. When adaptation was limited to fractions that initially presented violations, fewer new violations were introduced (2 mm: 60; 5 mm: 49).

**Conclusion:**

Daily-ART with 2 mm margins improved target coverage and reduced protocol violations, while larger margins increased OAR doses by enforcing full PTV coverage. Restricting daily ART to fractions with identified violations minimized the occurrence of newly introduced violations.

## Highlights

• A dose-protocol was created to evaluate dose distributions in head and neck cancer.
• Protocol was applied to compare violations across adaptive radiotherapy strategies.
• Daily adaptation (2 mm margins) improved target coverage and reduced violations.
• Daily adaptation (5 mm margins) increased organ doses and violations.
• Selective, protocol-driven adaptation minimized violations.

## Graphical abstract

**Figure:**
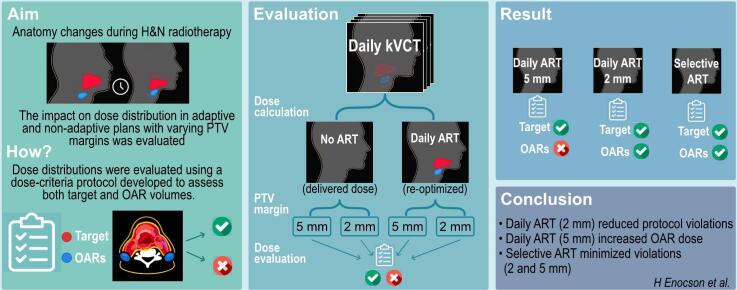

## Introduction

During the course of radiotherapy (RT), anatomical changes between treatment fractions, such as tumor shrinkage, weight loss, or organ motion, are common and can alter the planned/intended dose distribution. Such geometric uncertainties are addressed through robust immobilisation, appropriate safety margins expanding clinical target volumes (CTV) to planning target volumes (PTV) during plan optimisation, and image/surface-guided radiotherapy during treatment delivery. However, even with these safeguards, anatomical variations can cause discrepancies between the planned and delivered dose. To further account for potential anatomical variations, adaptive radiotherapy (ART) can be used, where the treatment dose and margins are modified based on the individual patient’s anatomical changes during the course of RT [1]. ART may be delivered via offline approaches between fractions (scheduled or ad hoc) or through online adaptation with the patient in-room [2].

Head and neck squamous cell carcinoma (HNSCC) is typically treated with long-course radiotherapy, often combined with surgery and/or systemic therapy according to international guidelines [3]. While several studies have investigated the potential benefits of ART in HNSCC in recent decades, its impact on clinical outcomes has not yet been established. Anatomical variations such as target shrinkage (including both primary and nodal volumes) [4], [5], [6], [7], [8], [9], parotid gland shrinkage [6], [9], [10], and patient weight loss [11] may compromise dose conformity and suggest potential benefits of ART. Studies reporting benefits in tumor control are few and inconclusive [12]. Several previous studies have evaluated ART for organ at risk (OAR) sparing, but results have been inconsistent. For example, parotid dose reductions have ranged from 0 to 10 Gy [5], [9], [13], [14], [15], but have not yet translated into reduced toxicity in clinical trials [16]. Several authors have suggested that margin reductions may be feasible with ART [17], [18], [19], [20]. In line with this, we previously found that margin reduction was necessary to achieve significant dose reductions to OARs in conjunction with ART [21].

According to published reviews, evidence on optimal ART strategies for HNSCC is limited by small patient cohorts [4], [12], which may lead to sample bias. Additionally, the literature presents a wide range of approaches, with variations in the number and timing of adaptations, PTV margins, and strategies for managing daily or accumulated dose deviations. Another important factor in ART is the workload associated with re-planning, which remains a major bottleneck in clinical implementation [2]. To address this, some studies have suggested that adaptation should be performed before the fourth week of treatment [10], [22], [23], and that limiting to three re-plans may be sufficient to keep OAR dose increases below 3  Gy [24]. Patient selection methods have also been explored as a way to reduce workload [25], [26], [27], [28] as well as various automatic selection strategies during treatment to identify fractions requiring replanning. This include triggers based on volumetric or density changes observed in verification imaging [28], [29], [30], or more advanced approaches using dose distribution constraints [31], [32], [33]. These dose-constraint protocols have been applied to evaluate non-adaptive plans, with limited studies comparing protocol adherence in both adaptive and non-adaptive scenarios.

Although an optimal ART strategy for HNSCC has yet to be clearly defined, survey data suggest that approximately 1 in 10 HNSCC patients require replanning during the course of treatment [34]. Moreover, while 55% of clinics report using ART for HNSCC, most rely on scheduled or ad-hoc adaptations, and only 10% employ predefined protocols with clear action thresholds [2]. These findings underscore the current lack of standardization and clinical guidance in ART practice.

Several studies have proposed dose-based criteria for ART decision-making. Barragán-Montero et al. [31] proposed an iterative framework for developing ART protocols and emphasized that deviations from the planned mean dose were more relevant for parallel OARs, whereas threshold exceedance was relevant for serial OARs, suggesting a 20% deviation from the dose constraint as an ART-trigger. Hunter et al. [35] reported that parotid dose deviations below 3.6  Gy were not associated with increased toxicity, suggesting it as a practical threshold for ART. Similarly, McCullenoch et al. [32] proposed that ART should be considered when delivered dose exceeds the planned dose by 15% of the dose constraint (typically 3–4.5  Gy depending on the OAR), and ± 7% deviation from the prescribed dose in D_95%,CTV_, in line with ICRU guidelines [36]. Several others [12], [24], [27] have used 3  Gy as a threshold for identifying HNSCC patients who may benefit from ART.

The aim of this study was to identify and evaluate a suitable protocol for objectively assessing dose distributions in RT for HNSCC. The study focused on different types and severities of dosimetric deviations that may prompt ART, aiming to evaluate both target coverage and OAR sparing. This protocol was applied to compare adherence between retrospectively simulated online adaptive and non-adaptive treatments using varying PTV margins.

## Materials/Methods

### Patients

A retrospective analysis was conducted on a consecutive cohort of 80 HNSCC patients (2170 treatment fractions) treated at Skåne University Hospital (Lund, Sweden) between August 2022 and May 2024 (Table 1). All patients received curative-intent RT using helical tomotherapy (HT) on a CT-linac (Radixact®, Accuray, Madison, WI). Patients with distant metastases were excluded. Five patients underwent a single re-plan during treatment due to anatomical or immobilisation changes, based on clinician’s assessment. Prescribed doses ranged from 60.0 to 68.0  Gy (2.0  Gy/fraction) to the primary tumor and involved nodal volumes (PTV-T and PTV-N), and 50.0 to 54.4  Gy to elective regions. Target volumes and OARs were contoured according to ICRU guidelines [37], [38]. All treatment plans used a uniform 5  mm CTV-PTV margin and a simultaneous integrated boost (SIB) technique, created in a commercial treatment planning system (RayStation v11A and v12A, Raysearch Laboratories, Stockholm, Sweden). Daily pre-treatment imaging was performed using fan-beam kVCT (ClearRT™, Accuray, Madison, WI), with the field of view (FOV) covering the entire target volume. Patient data collection and analysis were approved by the Swedish Ethical Review Authority (Dnr 2020–04164, Stockholm, Sweden).

**Table 1:** Study cohort and treatment characteristics.

Patient Characteristics	N	%
Total	80	100
Age (years)		
Median (range)	64.5 (41 to 84)	
>65	35	44
≤65	45	56
Sex		
Female	29	36
Male	51	64
BMI		
Median (range)	26.4 (18.0 to 34.1)	
Primary tumor site		
Oral cancer	19	24
Oropharynx	42	53
Salivary glands	8	10
Larynx	8	10
Hypopharynx	2	2
CUP	1	1
T stage*		
T0	2	2
T1	5	6
T2	34	43
T3	21	26
T4	18	23
Nodal Status		
N0	18	23
N1	35	44
N2	10	12
N2b	7	9
N2c	8	10
N3b	2	2
p16**		
Positive	39	91
Negative	4	9
Surgery (Reconstructive)***	28 (15)	35 (19)
Concomitant chemotherapy	17	21
Prescribed dose (Gy)		
68.0	57	71
60.0–66.0	23	29
Reirradiation	1	1
Re-planning during RT	5	6
Weight change during RT (kg)		
Median (range)	−2.4 (−11.7 to 3.7)	
Primary GTV volume (cc)		
Median (range)	5.9 (0 to 61.1)	
Nodual GTV volume (cc)		
Median (range)	12.7 (0 to 84.5)	
Total CTV volume (cc)		
Median (range)	222.7 (40.3 to 666.4)	

*Staging according to the AJCC 8th edition TNM classification. T0 cases included one CUP and one low-grade local recurrence of laryngeal cancer of laryngeal cancer not previously treated with radiotherapy. **p16 only relevant for oropharyngeal cancer and CUP. ***Surgical procedures which included reconstruction with either local or free flaps. BMI; Body mass index. CUP; Cancer of unknown primary. CTV; Clinical target volume.

### Synthetic CT-Based treatment simulation

A synthetic CT (sCT) was generated using deformable image registration (DIR) between the planning CT (pCT) and the daily fan-beam kVCT image. This process corrected for missing FOV in the daily kVCT by filling in omitted regions outside the scan range and aligned HU values to the pCT calibration curve. Contoured structures from the pCT were propagated onto the sCTs using DIR. All propagated structures were visually inspected and manually adjusted when necessary by a medical physicist, with support from a radiation oncologist.

To simulate the no-ART treatment, the clinical plan (5  mm CTV–PTV margin) and an additional plan with a 2  mm margin were recalculated on the sCTs for each fraction. In parallel, daily online ART treatments were simulated by generating plans using the same optimization parameters and constraints as the nominal plan (minimizing OAR doses while maintaining D_98,PTV_ ≥ 95% and D_98,CTV_ ≥ 98%). The processes of sCT generation, contour propagation, and treatment simulation were performed in RayStation (v2024A DTK, RaySearch Laboratories) and have been described previously [21]. For the patients who underwent re-planning during treatment, the no-ART scenario was modeled as if no re-planning had occurred. Consequently, for these patients, the no-ART scenario does not reflect the delivered treatment. Dose distributions from daily-ART and no-ART plans were deformed back to the pCT and accumulated, representing the total treatment.

### Dose-protocol creation and evaluation

Based on published literature and the DAHANCA treatment planning guidelines [39], a multidisciplinary team of medical physicists and radiation oncologists developed a dose protocol that categorizes ROIs into four groups, corresponding to four violation types, each with its own evaluation criteria:

1. **CTV volumes**: Delivered dose to 98% of the CTV must be equal to or greater than 95% of the prescribed dose.
2. **Serial OARs with MANDATORY constraints**: Delivered near-maximum dose (D_0.03cc_) or absolute maximum dose must remain below clinical threshold.
3. **Parallel OARs with SHOULD constraints**: Delivered mean dose must not exceed planned mean dose by more than 3  Gy.
4. **Other OARs with RECOMMENDED constraints**: Delivered near-maximum dose must not exceed defined threshold and planned dose by more than 3  Gy.

The complete set of ROI-specific criterium are summarized in Table 2. This protocol was applied to assess each daily dose distribution across all four scenarios; daily-ART and no-ART plans with both 5  mm and 2  mm CTV-PTV margins, and the corresponding total accumulated doses. For each patient and fraction, violations in the no-ART and daily-ART plans were compared separately for the 2 mm and 5 mm CTV-PTV margins. Violations were then classified as **resolved** (present in the non-ART plan but not in the daily-ART plan), **introduced** (absent in the no-ART plan but present in the daily-ART plan), or **persisting** (present in both the no-ART and daily-ART plans).

**Table 2:** Summary of protocol violation types, associated ROIs, evaluation metrics, and corresponding violation conditions. * Cochlea criteria was 40  Gy or 10  Gy with concomitant chemotherapy.

Violation Type	ROIs	Threshold (Gy)	Evaluation Metric	Violation Condition
1	CTV	–	D_98%_	D_98%_ < 95% of prescribed dose
2	Spinal Cord Brainstem Chiasm Optic Nerves RetinasBrain	48 54 54 54 4568	D_max_ D_0.03cc_ D_0.03cc_ D_0.03cc_ D_0.03cc_ D_0.03cc_	Delivered dose exceeds threshold
3	Parotid glands Larynx Submandibular glands Esophagus Oral Cavity Pharynx Constrictor muscles Lacrimal GlandsPituitary	–	Mean dose	Delivered mean dose exceeds the planned by more than 3 Gy
4	Brain outside PTV + 2 mm Cochlea Lens MandibleCarotid	60 40/10* 5 6840	D_1cc_ D_0.03cc_ D_0.03cc_ D_0.03cc_ D_0.03cc_	Delivered dose exceeds both the threshold and planned dose by more than 3 Gy

### Statistics

Wilcoxon signed-rank test was used to compare the number of protocol violations between daily-ART and no-ART plans. Mann-Whitney *U* test compared the number of fractions with protocol violations between patients with and without accumulated dose violations, for each strategy. Kruskal-Wallis tests were used to evaluate the relationship between the number of protocol violations and categorical patient characteristics and Spearman’s tests to evaluate the correlation between number of protocol violations and continuous patient characteristics, both assuming independent observations. Spearman’s correlation was used to evaluate the relationship between the mean external contour volume (per patient, relative to the pCT) and the number of violations, for each of the four scenarios). Similarly, Spearman’s correlation was used to investigate whether the number of violations for a specific ROI was associated with changes in that ROI’s volume relative to the pCT. The corresponding functions wilcoxon, kruskal, mannwhitneyu and spearmanr, from the scipy.stats module in Python 3.11.9 were employed to conduct these statistical tests. In this exploratory analysis, p-values less than 0.05 were considered statistically significant.

## Results

**Fig. 1:**
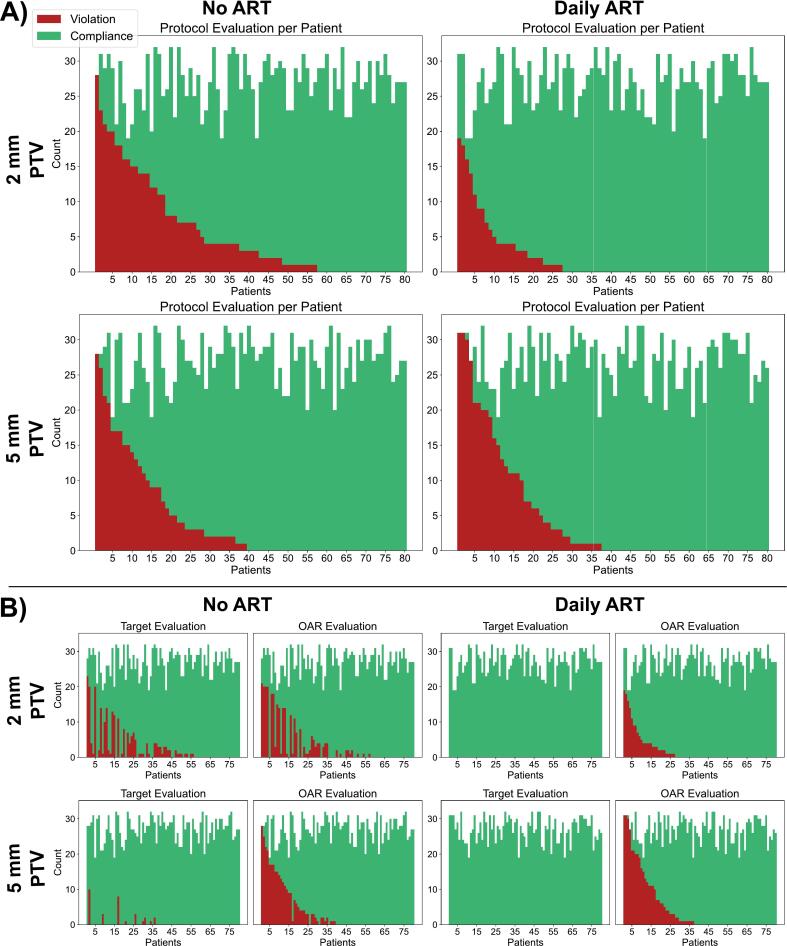
Number of fractions with protocol violations (red) and without violations (green) per patient across all treatment scenarios: No ART and Daily ART, with 2  mm and 5  mm CTV-PTV margin. A) shows total violations per patient and B) separate violations by type: target coverage or OAR constraint. Patients are ordered in descending number of violations within each scenario; patient numbers are therefore not consistent across scenarios. The plot should be interpreted as: a smaller red and larger green area represents a better, more robust strategy that should result in dosimetry more consistent with the approved treatment plan.

Fig. 1 show the distribution of protocol violations per patient across the four scenarios. For the 2  mm CTV–PTV margin, the no-ART scenario experienced significantly more violations (440, 20.3%, mean per patient = 5.5) compared to daily-ART (156, 7%, mean = 1.9; p < 0.001), for both target (mean = 2.85 vs. 0.0; p < 0.001) and OAR violations (mean = 3.39 vs. 1.9; p = 0.044). For the 5  mm margin, no significant differences were found in total (mean = 4.1, 15.1% vs. 4.69, 17.3%; p > 0.8) or OAR violations (3.86 vs. 4.69; p > 0.6) violations for no-ART and daily-ART respectively, while the number of target violations was significantly higher for no-ART (mean = 0.39 vs. 0.00; p = 0.007). The dose deviations associated with violations are presented in Supplementary Fig. S1.

**Fig. 2:**
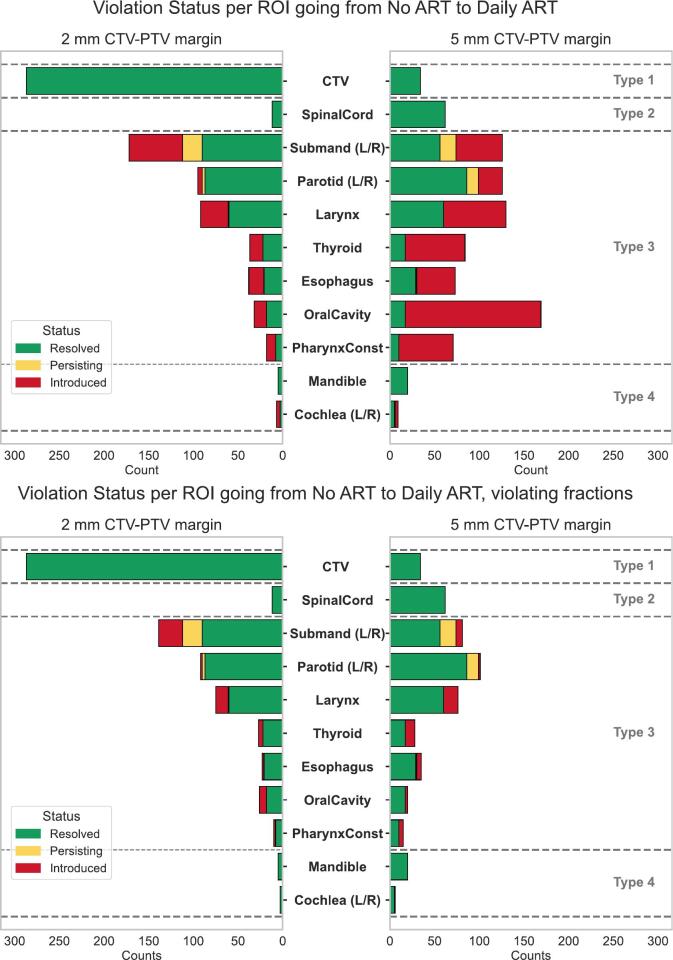
Visualization of the number of resolved (green), persisting (yellow), and introduced (red) protocol violations when transitioning from No ART to Daily ART, for both 2  mm and 5  mm CTV-to-PTV margin plans. The two bar plots on the left show the total number of violations across all fractions for No ART and Daily ART. The two bar plots on the right include only fractions with protocol violations in the No ART scenario, illustrating the effect of applying ART selectively to these fractions.

With 2  mm margin, transitioning from no-ART to daily-ART resulted in 610 resolved, 26 persisting, and 152 introduced violations. For the 5  mm margin, 391 violations were resolved, 32 persisted, and 472 were introduced. When restricting adaptation to fractions with protocol violations in the no-ART plan, the number of introduced violations decreased, to 60 for the 2  mm margin and 49 for the 5  mm margin, Fig. 2.

**Fig. 3:**
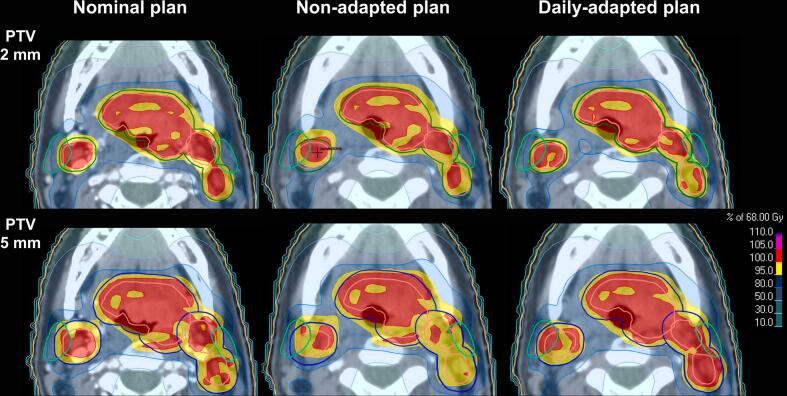
Example of parotid (light green) violations introduced by daily ART in a patient treated with a 5  mm CTV-to-PTV margin, which were not observed with a 2  mm margin. For each margin, dose distributions are shown for the nominal plan, the non-adapted plan, and the daily adapted plan. The PTV is outlined in blue (5  mm) and green (2  mm), and the CTV in pink. Plans are optimized to ensure that 95% of the prescribed dose is covered by the yellow isodose line.

Fig. 3 illustrates introduced violations for a representative patient, showing dose distributions for the nominal plan, the no-ART plan, and the daily-ART plan. Protocol violation was introduced in the parotid gland in the 5 mm ART-plan, despite adequate CTV coverage in the no-ART plan. The 2 mm ART-plan effectively restored CTV coverage. Several introduced violations in 5 mm ART plans originated from oral cavity violations, mostly in patients with oral cavity primaries and increased dose coinciding with adjustments improving PTV/CTV coverage.

Mann-Whitney U tests showed significantly higher numbers of daily violations in patients with accumulated violations across all four strategies (U = 463.5–798.5, p = 0.00058–0.0126). For violation type 3, a significant difference was observed between patients with and without accumulated type 3 violation in the 5 mm no-ART plans (U = 24.0, p = 0.011). No significant differences were observed for violation types 1, 2, and 4, partly due to limited variation.

Spearman’s test showed a significant negative correlation between the mean relative volume of the external ROI and the number of violations in the no-ART plans. The no-ART 2 mm and no-ART 5 mm groups showed correlation coefficients of −0.227 (p = 0.0427) and −0.262 (p = 0.0188), respectively. No significant correlations were observed for either of the daily-ART plans (p > 0.6). Similarly, for several OARs, both no-ART groups demonstrated negative associations with the corresponding relative volumes. Significant correlations were found for the parotid (L/R) (r = –0.451, p < 0.001; r = –0.421, p < 0.001) and submandibular (L/R) (r = –0.433, p < 0.001; r = –0.326, p = 0.004) for the no-ART 2 mm and 5 mm margin plans, respectively. Target coverage violation and CTV volume change did not show significant correlations in any of the groups (p > 0.1). Full correlation results are provided in Supplementary Table S1.

Spearman’s correlation showed significant associations between number of violations and CTV volume, primary GTV volume, prescribed dose, and weight loss during treatment in the no-ART 2 mm and 5 mm plans. These correlations were generally weaker and non-significant in the corresponding daily-ART plans. For the no-ART 2 mm and 5 mm plans, Kruskal–Wallis tests indicated significant differences in violation counts across several categorical characteristics, with higher numbers of violations in patients who underwent surgery, with oropharyngeal or laryngeal primary tumors, and concomitant chemotherapy (Supplementary Fig. S2 and Table S2).

## Discussion

In this study, we have shown that the introduction of daily ART may improve target coverage, but at the cost of increased doses to OARs in some patients. Thus, the benefit of ART for HNSCC patients is questionable when large PTV margins are used. The benefit of daily ART appears to be more pronounced when reduced PTV margins and/or protocol-driven adaptation are applied. We developed and evaluated a protocol for assessing dose distributions in RT for HNSCC. Relevant criteria were defined for target and OARs volumes. For parallel OARs, ART may be beneficial regardless of proximity to clinical thresholds, as complication generally increases continuously with dose [40]. For serial OARs, reducing dose below the threshold provides limited additional clinical benefit [31]. These distinctions guided us in identifying clinically relevant violations. Here, we demonstrated that this protocol can be useful in guiding online ART workflows but may also be useful in offline workflows by promoting consistency across patients, rather than ad-hoc replanning.

Of the 2170 evaluated fractions, 15% and 20% showed protocol violations for the 5 mm and 2 mm no-ART plans, respectively, consistent with Barragan-Montero et al. 2023 [31], where physician-triggered ART occurred in ∼ 20% of fractions and protocol-triggered ART slightly more frequently. Further, Barragan-Montero evaluated PTV coverage rather than CTV coverage, which may contribute to a higher triggering frequency.

Plans with a 2  mm margin benefited from daily-ART, which reduced the number of violations compared to no-ART (Fig. 1, Fig. 2). While 2 mm no-ART plans initially showed more CTV violations than larger-margin plans, these were effectively resolved with daily-ART. The reduced margin inherently limited OAR dose exposure [21], while ART consistently improved CTV coverage. When ART was applied selectively, only on fractions showing violations in the no-ART plan, the number of introduced violations decreased for both the 2  mm and, more prominently, the 5  mm margin plans (Fig. 2). This suggests that combining reduced margins with a selective ART strategy, rather than uniform daily-ART, may offer balance between target coverage and normal tissue sparing, while minimizing unnecessary replanning. It should be noted that this reflects a triggered online-ART scenario; whether similar benefits could be achieved using a triggered offline strategy for subsequent fractions remain unknown and was not addressed in this study.

For the 5  mm margin plans, daily-ART did not reduce the overall number of protocol violations (Fig. 1). In fact, more violations were observed in the daily-ART plans compared to no-ART, although not statistically significant. As shown in Fig. 2, violations were mainly of type 3, for which adaptation both resolved and introduced violations. This primarily reflects the optimizer enforcing full PTV coverage even when CTV coverage was already sufficient, leading to increased dose to nearby OARs, a pattern not observed in the 2  mm scenario. Fig. 3 illustrates an example of this: the 5  mm ART plan optimized PTV coverage despite adequate CTV coverage in the no-ART plan, whereas the 2  mm ART plan effectively recovered CTV coverage.

We observed significant correlations between OAR volume changes and protocol violation in the no-ART plans. Specifically, reductions in external volume were associated with more violations, and decreases in parotid and submandibular gland volumes correlated with corresponding ROI-specific violations. These correlations were not observed in the daily-ART plans, suggesting that adaptation may mitigate dosimetric consequences of anatomical variation. These findings support the potential use of anatomical changes as predictive indicators or triggers for adaptive planning. Moreover, the significant associations between higher numbers of violations and larger treatment volumes, larger primary tumors, weight loss, oropharynx and larynx cancers, prior surgery, and concomitant chemotherapy in the no-ART plans, but not in the daily-ART plans, suggest that these patient groups may benefit more from ART.

This work should be viewed in light of certain limitations. The choice of protocol criteria affects our results, although changing thresholds would likely yield similar patterns, based on the violating doses presented in Supplementary Fig. S1. In addition, the choice of PTV margins in the plans evaluated also affects the results, although we believe similar effects would be observed when comparing different margin sizes. When evaluating daily adaptation, delivered doses were not accumulated from previous fractions up to the fraction being evaluated, as done in some studies [24], [32]; instead, each daily plan was assessed independently, assuming that the entire treatment was delivered according to that day's anatomy. However, significantly higher numbers of daily violations were observed in patients with violations in the accumulated dose, supporting the relevance of daily assessments. Also not the, accumulated doses are inherently uncertain. In our study, DIR inaccuracies led to the need for manual re-contouring of some structures, and these uncertainties propagate into uncertainties in dose deformation/accumulation. This limitation is well recognized, and becomes more prominent in dose deformation compared to contour propagation [41]. While deformable dose accumulation is sufficiently accurate for evaluating target coverage in head and neck cancer, larger uncertainties have been reported in high-gradient regions, particularly for OARs near the target [42], [43]. For this reason, we considered daily dose distributions to provide a more reliable and interpretable basis for evaluating plan quality in this study.

In conclusion, daily-ART improves plan quality and decreases the number of dose-protocol violations for plans with small margins (2 mm). For larger margins (5 mm), we observe an increased number of violations by enforcing full PTV coverage at the expense of OAR doses. Importantly, limiting adaptation to only fractions where protocol criteria were unmet, minimized introduced violations and unnecessary replanning. Combining reduced margins with a protocol-driven strategy may offer a balance between target coverage, normal tissue sparing, and treatment efficiency.

## CRediT authorship contribution statement

**Hedda Enocson:** Writing – original draft, Visualization, Methodology, Investigation, Formal analysis, Data curation. **André Haraldsson:** Writing – review & editing, Supervision, Methodology, Conceptualization. **Per Engström:** Writing – review & editing, Supervision, Methodology. **Sofie Ceberg:** Writing – review & editing, Supervision, Methodology. **Maria Gebre-Medhin:** Writing – review & editing, Validation, Supervision, Methodology. **Gabriel Adrian:** Writing – review & editing, Validation, Supervision. **Per Munck af Rosenschöld:** Writing – review & editing, Supervision, Resources, Project administration, Funding acquisition, Conceptualization.

## Declaration of competing interest

The authors declare that they have no known competing financial interests or personal relationships that could have appeared to influence the work reported in this paper.

## Appendix A Supplementary data

Supplementary Data 1 Extended dose analyses and statistical results.
