# Supplementary material for: Daily adaptive radiotherapy in head and neck squamous cell carcinoma can negatively impact organ at risk dosimetry
Source: Clin Transl Radiat Oncol. 2026 May 30;59:101205. doi: 10.1016/j.ctro.2026.101205 (PMC13251744; doi:10.1016/j.ctro.2026.101205)
Supplement: Supplementary Data 1 — Extended dose analyses and statistical results. [file mmc1.pdf]

Supplementary

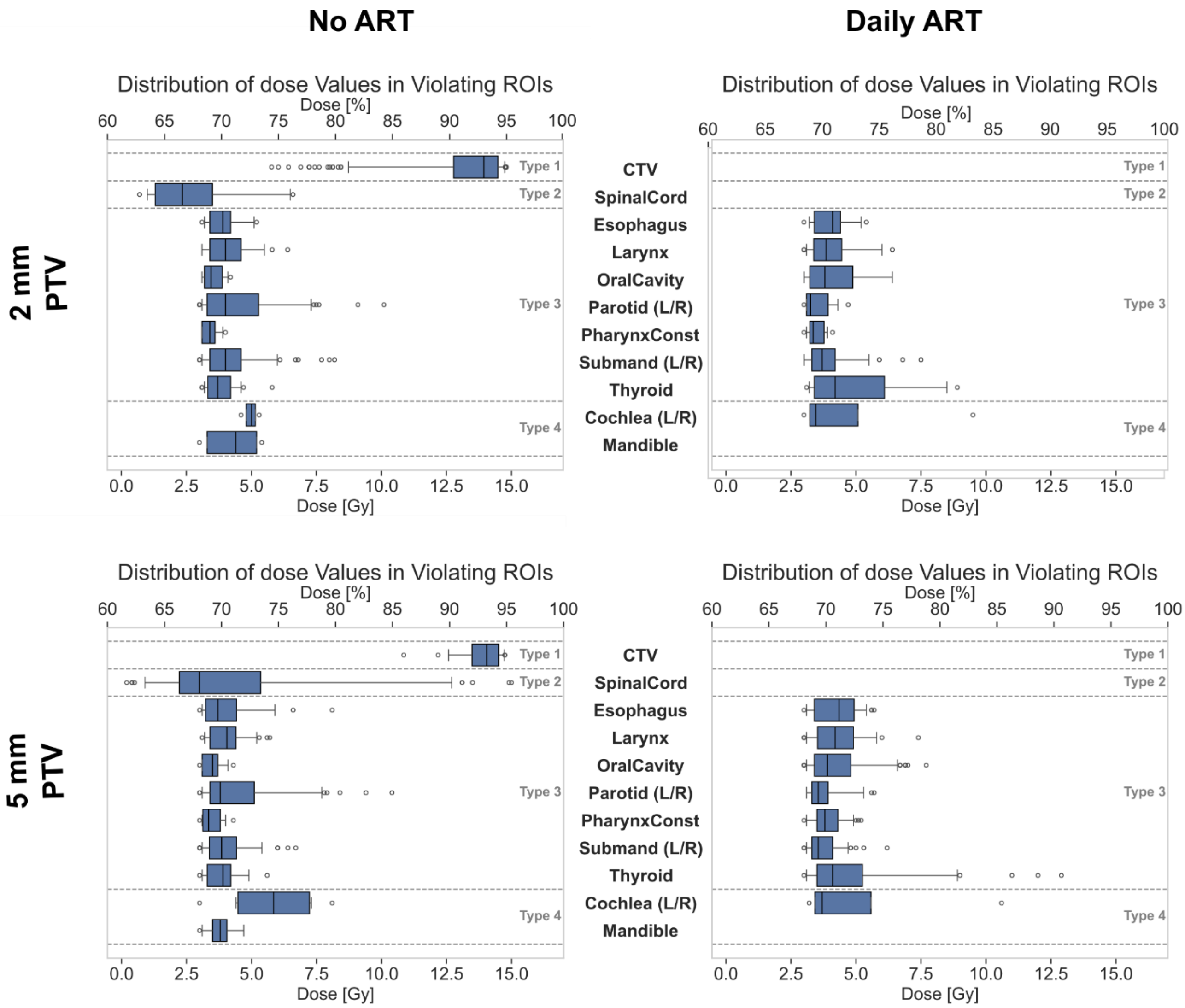

Figure S1. Doses for fractions that violated protocol criteria across the four strategies. For violation type 1, the dose is presented as a percentage of the prescribed dose, whereas for the remaining violation types the dose is presented as deviations from the planned dose

Table S1. Spearman's correlation between ROI violation and ROI volume change. No failures were observed in the ART plans for CTV.

ROI	No ART 5mm	Daily ART 5mm	No ART 2mm	Daily ART 2mm
Parotid (L/R)	-0.42 **(p<0.001)**	-0.13 (p=0.24)	-0.45 **(p<0.001)**	-0.11 (p=0.36)
OralCavity	-0.06 (p=0.61)	0.18 (p=0.11)	0.01 (p=0.93)	0.27 **(p=0.018)**
Submand (L/R)	-0.33 **(p<0.001)**	-0.14 (p=0.23)	-0.43 **(p<0.001)**	-0.33 **(p=0.0034)**
Esophagus	-0.08 (p=0.53)	-0.01 (p=0.93)	-0.16 (p=0.21)	-0.06 (p=0.64)
CTV	0.10 (p=0.36)	*-not listed-*	0.12 (p=0.29)	*-not listed-*
Larynx	-0.21 (p=0.09)	0.02 (p=0.88)	-0.28 **(p=0.019)**	-0.17 (p=0.17)
Thyroid	0.05 (p=0.84)	0.04 (p=0.87)	0.05 (p=0.84)	-0.11 (p=0.67)
PharynxConst	0.18 (p=0.21)	-0.14 (p=0.35)	0.18 (p=0.21)	-0.23 (p=0.10)

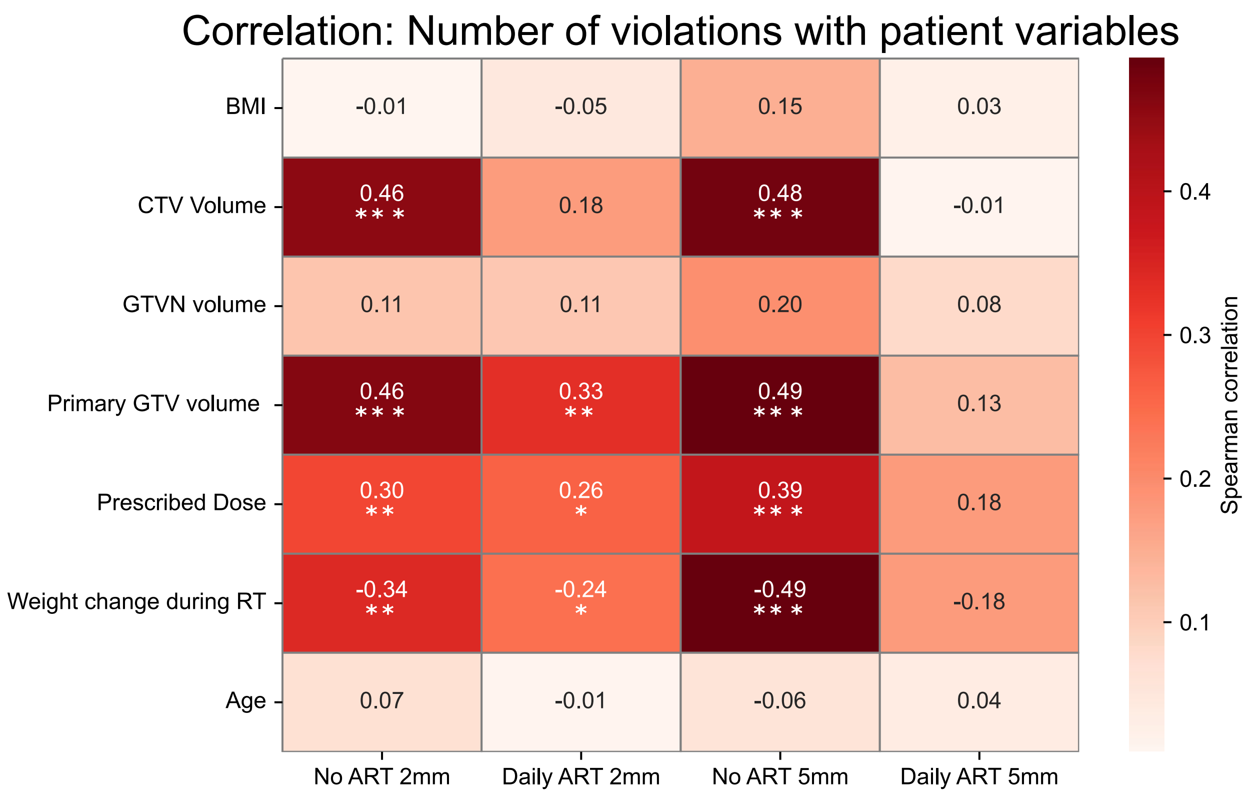

*Figure S2. Spearman correlation of number of violations and numerical patient variables for the four strategies; no ART 2 mm, daily ART 2 mm, no ART 5 mm, daily ART 5 mm. Color indicate correlation in absolute numbers and the correlation coefficient and significance is written for each case. * indicate p< 0.05, ** p<0.001, *** p<0.0001.*

*Table S2. Kruskal–Wallis statistics for all categorical variables with significant correlation with of number of violations for each scenario along with mean number of violations per variable group. The number of patients in each variable group can be seen in patient characteristics in Table 1. * Reconstructive surgery, **p16 only relevant for oropharyngeal cancer and CUP.*

Scenario	Patient Variable	H statistic	P Value	Group Means
ART 2 mm	Surgery	9.006	0.01	No: 2.69<br>Yes: 1.38<br>Yes (rec*): 0.07
No ART 2 mm	Surgery	15.072	0.0005	No: 7.04<br>Yes: 4.31<br>Yes (rec*): 1.2
No ART 5 mm	Surgery	17.238	0.0002	No: 5.65<br>Yes: 3.5<br>Yes (rec*): 0.0
No ART 2 mm	p16	9.526	0.009	Negative: 4.75<br>Positive: 7.49<br>Not rel**: 3.49
No ART 5 mm	p16	7.304	0.03	Negative: 7.5<br>Positive: 5.37<br>Not rel**: 2.67
No ART 2 mm	Primary tumor site	16.597	0.005	Oral Cancer: 2.89<br>Oropharynx: 6.93<br>Salivary glands: 1.38<br>Larynx: 7.5<br>CUP: 20.0<br>Hypopharynx: 1.5
No ART 5 mm	Primary tumor site	12.476	0.03	Oral Cancer: 1.84<br>Oropharynx: 5.66<br>Salivary glands: 0.71<br>Larynx: 6.5<br>CUP: 2.0<br>Hypopharynx: 2.0
No ART 2 mm	Concomitant chemotherapy	13.759	0.0002	No: 3.97<br>Yes: 11.18
No ART 5 mm	Concomitant chemotherapy	19.785	<0.0001	No: 2.36<br>Yes: 10.94
